# Molecular Regulation of Copper Homeostasis in the Male Gonad during the Process of Spermatogenesis

**DOI:** 10.3390/ijms21239053

**Published:** 2020-11-28

**Authors:** Sylwia Herman, Paweł Lipiński, Mateusz Ogórek, Rafał Starzyński, Paweł Grzmil, Aleksandra Bednarz, Małgorzata Lenartowicz

**Affiliations:** 1 Department of Genetics and Evolution, Institute of Zoology and Biomedical Research, Jagiellonian University, Gronostajowa 9, 30-387 Kraków, Poland; sylwia.herman@doctoral.uj.edu.pl (S.H.); m.ogorek1990@gmail.com (M.O.); pawel.grzmil@uj.edu.pl (P.G.); a.j.bednarz@doctoral.uj.edu.pl (A.B.); 2 Department of Molecular Biology, Institute of Genetics and Animal Biotechnology, Polish Academy of Sciences, 05-552 Magdalenka, Jastrzębiec, Poland; p.lipinski@igbzpan.pl (P.L.); r.starzynski@igbzpan.pl (R.S.)

**Keywords:** spermatogenesis, gametes production, copper, testis, CTR1, ATP7A, ATP7B

## Abstract

Owing to its redox properties, copper is a cofactor of enzymes that catalyze reactions in fundamental metabolic processes. However, copper–oxygen interaction, which is a source of toxic oxygen radicals generated by the Fenton reaction, makes copper a doubled-edged-sword in an oxygen environment. Among the microelements influencing male fertility, copper plays a special role because both copper deficiency and overload in the gonads worsen spermatozoa quality and disturb reproductive function in mammals. Male gametes are produced during spermatogenesis, a multi-step process that consumes large amounts of oxygen. Germ cells containing a high amount of unsaturated fatty acids in their membranes are particularly vulnerable to excess copper-mediated oxidative stress. In addition, an appropriate copper level is necessary to initiate meiosis in premeiotic germ cells. The balance between essential and toxic copper concentrations in germ cells at different stages of spermatogenesis and in Sertoli cells that support their development is handled by a network of copper importers, chaperones, recipient proteins, and exporters. Here, we describe coordinated regulation/functioning of copper-binding proteins expressed in germ and Sertoli cells with special emphasis on copper transporters, copper transporting ATPases, and SOD1, a copper-dependent antioxidant enzyme. These and other proteins assure copper bioavailability in germ cells and protection against copper toxicity.

## 1. Introduction

Copper (Cu) is a trace element necessary for the growth and development of all living organisms and is the third most abundant trace element in the body after iron and zinc [1]. In an organism, this reactive element can exist in two oxidation states: as a reduced, cuprous (Cu^+^) and as an oxidized, cupric (Cu^2+^) ion [2]. The extensive range of redox potentials of copper and its capacity to participate in one-electron transfer reactions determine the biological activity and function of this microelement. Owing to its redox properties, copper is a cofactor for more than 30 enzymes. Many Cu-dependent enzymes catalyze reactions used in fundamental metabolic processes including respiration (cytochrome c oxidase), detoxication of oxygen free radicals (superoxide dismutases: SOD1 and SOD3), connective tissue formation (lysyl oxidase), neurotransmitter synthesis (dopamine ß-hydroxylase and peptidylglycine-amidating monooxygenase, PAM), pigment production (tyrosinase), and iron metabolism (ceruloplasmin and hephaestin) [3,4,5]. Copper also takes part in the processes of myelination and regulation of the circadian rhythms, and is necessary for angiogenesis and coagulation [6]. Copper deficiency leads to the impaired function of these enzymes and in consequence to disturbances in the key metabolic processes. Conversely, by means of the same redox activity, copper can generate an extremely harmful hydroxyl radical (·OH) through the Fenton reaction [7], which may cause oxidative damage to proteins and nucleic acids, lipid peroxidation, and enzyme inhibition. For this reason, excess copper is highly toxic to organisms [2,3,7]. To deal with this dual nature of copper, tightly regulated molecular mechanisms have evolved and operate in organisms to ensure its bioavailability and to reduce its toxicity. The balance between essential and toxic copper concentrations at both cellular and systemic levels is maintained by a complex network of proteins involved in the regulation of copper uptake, transport, utilization, storage, and excretion.

## 2. An Outline of Systemic Copper Metabolism in Mammals

In adult mammals, copper is predominantly absorbed in the duodenum and small intestine and is transported to the blood from enterocytes. In adult humans, the average daily intake of copper ranges from 0.8 to 3 mg [3,4,8]. In the blood, absorbed copper is bound to albumin, histidine, or glutathione, and is then transported via the portal vein into the liver, a central organ that maintains copper homeostasis [3,4]. In hepatocytes, copper is bound to ceruloplasmin (CP) and is again excreted to the circulatory system, and with blood redistributed to other organs and tissues [9,10]. CP is a main copper carrier in the blood, transporting this microelement to various organs and tissues. Approximately 90% of serum copper occurs in the complex with CP, while the remaining 10% is bound to albumin or amino acids [4]. Copper, when delivered to various organs, is utilized in metabolic processes by Cu-containing enzymes. Although various tissues differ in copper requirements, the set of proteins regulating copper distribution within the cells is thought to be the same in all tissues. On the other hand, excess cytosolic copper in hepatocytes is expelled into the bile and removed from the organism in feces. Only 2% of copper is removed from the body by the kidneys in urine [4,11,12].

## 3. Cellular Copper Uptake, Transport, and Utilization Are Orchestrated by a Closely Integrated Network of Proteins

**Figure 1:**
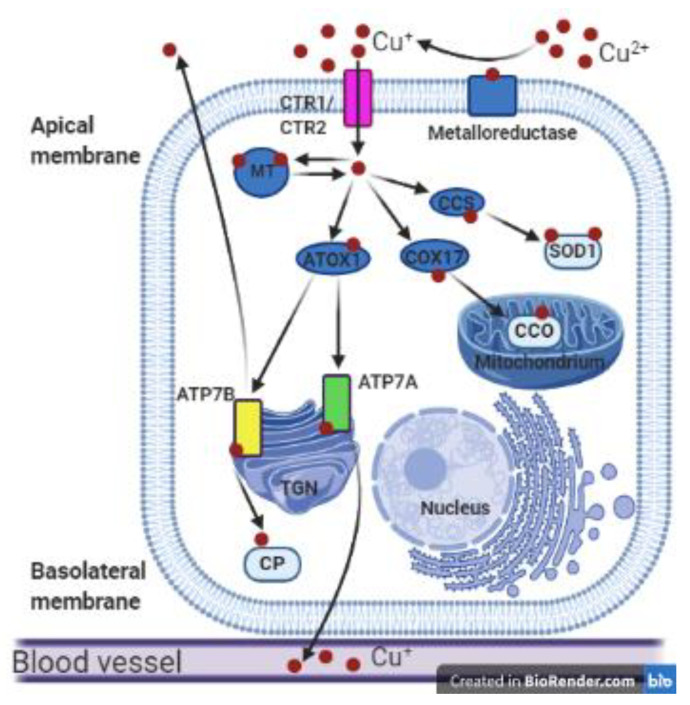
Copper metabolism in polarized mammalian cells. Cupric ions (Cu^2+^) are reduced to cuprous ions (Cu^+^) by metaloreductase and can then be transported into the cell by the transmembrane transporters CTR1 or CTR2. In the cytosol, Cu^+^ ions can be reversibly bound by metallothionein (MT) and stored in the Cu-MT complex. In the cytoplasm Cu^+^ ions are bound by cytosolic metallochaperones: copper chaperone for superoxide dismutase 1 (CCS), COX17, and ATOX1, which deliver copper to various proteins such as superoxide dismutase 1 (SOD1), cytochrome c oxidase (CCO), ATP7A, and ATP7B. As a result, copper is delivered to different cellular compartments such as the cytoplasmic reticulum, mitochondria, or the Golgi apparatus. The ATP7A and ATP7B proteins located in the trans-Golgi network (TGN) incorporate Cu^+^ ions into the apoenzymes. ATP7B can transfer copper to apo-ceruloplasmin (CP) to form holo-ceruloplasmin. Then copper in the complex with ceruloplasmin can be transported to the blood vessels. Cu-transporting ATPases can remove the excess copper outside the cell: ATP7A across the basolateral membrane and ATP7B across the apical membrane of polarized cells. This figure was created with BioRender.com.

Over the last three decades, a remarkable increase has been made in our understanding of the molecular mechanisms of copper homeostasis, as well as the structure and function of Cu-containing proteins. A real breakthrough was the discovery in 1993 of ATP7A and ATP7B proteins: Cu-transporting ATPases [13,14,15,16]. Currently, we know that cellular copper homeostasis requires a series of copper importers, carriers, chaperones, recipient proteins, and exporters to achieve the essential level of this biometal and prevent its toxicity (summarized in Figure 1). Cellular copper uptake, transport, and utilization are coordinated by a closely integrated network of three groups of proteins.

The first group comprises copper transporters belonging to the CTR/SLC31 (copper transporter) family: CTR1 and CTR2 proteins that are involved in copper uptake and intracellular distribution. In mammalian cells, copper import is primarily mediated by the high-affinity copper membrane transporter CTR1 (more than 80% of intracellular copper import is CTR1-dependent). Although CTR1 is ubiquitously expressed in tissues, its expression is especially high in the liver, kidneys, small intestine, ovaries, testes, and heart [17,18,19,20,21,22]. CTR2, a second cellular copper transporter, exhibits exclusively intracellular localization. CTR2 is localized in the membranes of the vacuoles, vesicles, endosomes, and lysosomes of mammalian cells [17,20,23]. The major role of CTR2 is to facilitate copper release from degraded cuproenzymes in lysosomes and to transport it back into the cytosol for reutilization [17,20].

The second group comprises metallochaperones, cytosolic copper transporters that bind Cu ions and deliver them to the cellular organelles to prevent possible participation of Cu ions in the Fenton reaction [7,18]. These metallochaperones include among others an antioxidant 1 protein (ATOX1), which binds Cu^+^ ions and transports them from the Golgi apparatus to ATP7A and ATP7B proteins [3,7,24]. ATOX1-ATP7A and ATOX1-ATP7B interactions are essential for intracellular copper translocation and trafficking because ATOX1 also regulates the catalytical activities of both ATPases [24,25]. The copper chaperone for superoxide dismutase 1 (CCS) is a ubiquitously expressed cytosolic metallochaperone, delivering Cu ions to Cu,Zn-SOD1 molecules in the cytoplasmic reticulum. By means of this function, CCS is responsible for the conversion of apo-SOD1 to the active holoenzyme [26,27,28,29]. Cytochrome c oxidase copper chaperone (COX17) is a metallochaperone that binds Cu ions and transports them to mitochondria, where they are bound to subunits of cytochrome-c oxidase, an enzyme of complex IV of the mitochondrial respiratory chain. COX17 is a small, hydrophilic protein located in the cytoplasm and mitochondrial intramembrane space. COX17 is expressed in all tissues [30,31].

The third group comprises P-type Cu-transporting ATP-ases (ATP7A and ATP7B), localized in the trans-Golgi network (TGN). In the Golgi apparatus, ATP7A and ATP7B proteins transfer copper, using the energy of ATP hydrolysis, into the lumen of the secretory pathway where this metal is incorporated into the active sites of the Cu-dependent enzymes. Both ATP7A and ATP7B are also involved in the ATP-dependent transport of Cu ions across the plasma or intracellular membranes [3,17,25,32]. ATP7A and ATP7B are encoded by different genes localized in separate chromosomes. In humans and laboratory rodents, ATP7A protein is encoded by the X-linked *ATP7A* (*Atp7a* in rodents) gene and its expression has been reported in nearly all cells in the body [13,14,15]. For this reason, *ATP7A* is considered to be a housekeeping gene. Nevertheless, its expression differs among the cells and tissues and is age-dependent [3,9,10,33]. The highest expression levels have been found in the brain, kidney, small intestine, and heart [3,34,35,36]. In humans, lack of ATP7A activity caused by a mutation in the *ATP7A* gene leads to severe metabolic syndrome—Menkes disease [1].

ATP7B is encoded by the autosomal *ATP7B* gene. In humans the *ATP7B* gene is located on chromosome 13, in mice on chromosome 8 [16,37,38]. In mammals, ATP7B protein is primarily synthesized in the liver in the hepatocyte, but its expression has been also reported in other tissues such as the placenta, mammary gland, eye, lung, and brain [3,16,38]. ATP7B protein in hepatocytes is responsible for copper binding to apo-ceruloplasmin, resulting in the formation of the redox-active holo-ceruloplasmin, responsible for the transport of copper into the bloodstream. ATP7B also participates in the excretion of excess copper to the bile [39]. Besides the liver, ATP7B is also responsible for the return transport of copper from the placenta to the maternal compartment, to prevent excess copper accumulation in the fetus [40,41]. During lactation in mammary gland cells, ATP7B expression exhibits a granular, diffuse cytoplasmic pattern, and participates in copper export to the milk [42]. Mutation in the *ATP7B* gene results in disturbances in copper binding to ceruloplasmin (CP) by the ATP7B protein and leads to copper accumulation in the liver up to the toxic level in patients with Wilson disease [3,43]. In cells, ATP7A and ATP7B are found predominantly in membranes of the TGN and endocytic vesicles, and to a lesser extent at the plasma membrane. ATP7A and ATP7B circulate between these compartments in response to changes in intracellular copper levels. The Cu-dependent regulation of intracellular localization of ATPases determines the fate of this metal, which is either used for the biosynthesis of Cu-dependent enzymes in the secretory pathway or is sequestered in or released from vesicles into the extracellular milieu [25,38]. By means of different mechanisms, both ATP7A and ATP7B prevent toxic copper accumulation by expelling Cu ions from the cells. When the cells are exposed to increased copper concentrations, ATP7A moves to the cytoplasm and plasma membrane (in polarized cells to the basolateral membrane) [17,32,38,44]. Under the same conditions, ATP7B is transported to the plasma membrane in vesicles (in polarized cells to the apical membrane) [2,25,38]. It has been proposed that this Cu-induced trafficking of both ATPases is fundamental for maintaining cellular copper homeostasis [3]. Another important role of ATP7A involves delivering of Cu^+^ ions to the secretory pathway where they are incorporated into the Cu-dependent enzymes such as lysyl oxidase, tyrosinase, dopamine-β-hydroxylase, peptidylglycine-α-amidating monooxygenase, and extracellular dismutase (SOD3) [3,45,46].

## 4. Dysfunction of Spermatogenesis in Both Copper Overload and Deficiency

Although our knowledge of the molecular regulation of copper metabolism has dramatically increased over the past 30 years, the precise molecular mechanisms of copper handling in the male reproductive system are still poorly understood. Moreover, available clinical data urgently wait for a better explanation of the regulation of copper acquisition and distribution within cells of the male reproductive system. Results of numerous human and animal studies indicate that both testis structure and the process of spermatogenesis (production, maturation, motility, and fertilizing capacity of the spermatozoa) are affected in response to increased copper concentration [47,48,49,50,51,52,53,54]. It has been also reported that seminal plasma copper concentrations in oligozoospermic, asthenozoospermic, and azoospermic patients are significantly higher than in normozoospermic individuals [55]. Even in infertile men, exposure to high environmental copper concentration is associated with increased oxidative stress manifested by the increased total oxidant value in the seminal plasma [56]. Clinical manifestations associated with copper poisoning such as seminiferous tubule epithelium degradation [48,54], reduction in spermatozoa number [49], decreased spermatozoa motility [48,56], acrosome reaction inhibition [57], and increased apoptosis have been reported to reduce male fertility [47,48]. Moreover, decreased testosterone levels have been noticed in rats supplemented with a high copper diet [49]. In young male mice injected with small amounts of cupric chloride (CuCl_2_), the development of gonads was delayed in comparison to control mice [48]. In addition, in infertile and subfertile men, blood serum and seminal plasma copper concentrations were significantly higher than those in the fertile controls [28,53,54]. Conversely, there is strong evidence showing that Cu-deficient ram, rat, mouse, and goat males produce ejaculates of lower volume, lower spermatozoa concentration, poorer spermatozoa motility, and morphology. Histological analyses have demonstrated that the seminiferous tubules of Cu-deficient animals are less developed. This was mainly explained by the inactivation of the function of the Sertoli cells [48,50,58,59,60,61]. All these data indicate that copper concentration and metabolism in male gonads must be very tightly regulated, starting from puberty and continuing progressively throughout adult life. Studies using the mouse model showed that in adult males, copper concentration in the testes (0.94 μg/g wet tissue) [48] is lower in comparison with other organs such as the brain (1.5 μg/g) [62], kidney (4.4 μg/g), and liver (4.94 μg/g) [10,12].

## 5. High-Affinity Copper Proteins from the CTR Family Facilitate Copper Transport to Both Sertoli and Germinal Cells

**Figure 2:**
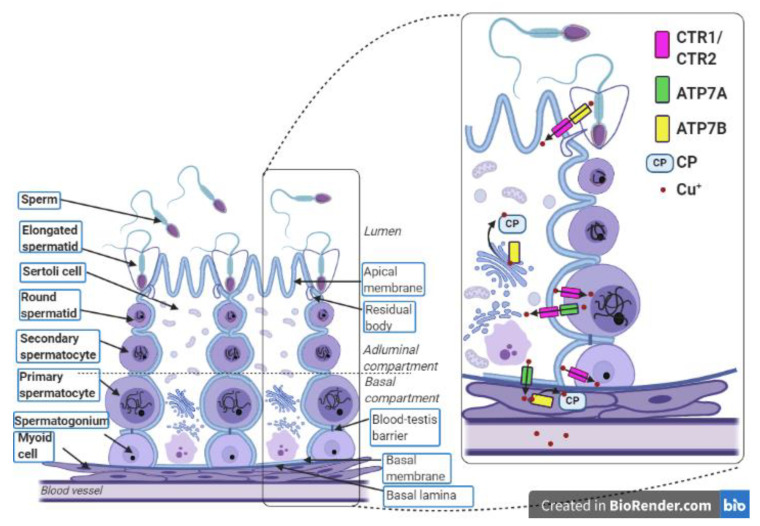
Localization of copper-related proteins in the seminiferous tubule and myoid cells. In the testes, gametes are produced during the multi-step process called spermatogenesis, which covers a complex network of regulations that occur in the seminiferous tubules. Each seminiferous tubule is surrounded by a few layers of myoid cells that border on blood vessels. The seminiferous epithelium contains the Sertoli cells and the germ cells and lies on the basal lamina. The lumen of the seminiferous tubule is filled with seminal fluid. The Sertoli cells are polarized cells that reach the lumen of the seminiferous tubule and support the individual stages of developing germ cells. Sertoli cells are linked by tight junctions that form a blood–testis barrier. This divides the seminiferous epithelium into the adluminal and basal compartments and allows Sertoli cells to control the environment for developing late germ cells. The first stages of the germ cell line are spermatogonia, which lie on the basal lamina. Above them, primary and secondary spermatocytes are successively situated. The next stages are round spermatids, which transform into elongated spermatids. After absorption of residual bodies, Sertoli cells release the elongated spermatids into the seminiferous tubule lumen where these spermatids become sperm. The transport of Cu ions during the process of spermatogenesis occurs between germ cells, Sertoli cells, and myoid cells. Spermatogonia, primary spermatocytes, and Sertoli cells absorb copper using CTR1. The excess copper is exported from the primary spermatocytes and Sertoli cells through the ATP7A protein. Presumably, a similar function is performed by ATP7B in the elongated spermatids. Removed Cu^+^ ions are imported to Sertoli cells through CTR1. In Sertoli cells and myoid cells, copper is delivered to the ceruloplasmin via ATP7B. In the complex with ceruloplasmin, Cu^+^ ions leave the myoid cells and enter the bloodstream. This figure was created with BioRender.com.

Copper is transported from the blood to the gonads and is distributed all over the organ through the capillary vessels. Up to now, it is unclear how copper is delivered from capillary vessels to seminiferous tubules. Importantly, seminiferous tubules are penetrated by neither blood nor lymph vessels. In rodents, seminiferous tubules are closely surrounded by tunica propria composed of fibroblasts and the myoid cell layer (shown in Figure 2) [63]. Many transporters expressed either in Sertoli cells or germinal cells act to facilitate the influx and/or efflux of metabolites and create a suitable environment for spermatogenesis [63,64]. The results of our recent study indicate an age-dependent regulation of the expression of copper transporter genes *Slc31a1* and *Slc31a2* (encoding CTR1 and CTR2 proteins, respectively) in the mouse immature and mature male gonads [65]. CTR1 transports copper in the form of Cu^+^ ions in an ATP-independent manner using conserved methionine residues located in the N-terminal domain. Two-dimensional electron microscopy studies have demonstrated that CTR1 forms a pore for the movement of Cu^+^ ions across membranes by the formation of homotrimers within the cell membrane [20,21]. Studies on mouse models provide evidence that within the seminiferous tubules of adult males, high-affinity copper transporter CTR1 mediates copper transport to both Sertoli cells and germinal cells [19,64,65]. In the cross-section of the testis, the expression of CTR1 has been detected in spermatogonial cells and primary spermatocytes [64,65]. Therefore, it seems that CTR1 plays a major role in the supply of copper to premeiotic and early meiotic germinal cells. In addition, our findings revealed that in the mouse testis, expression of the *Slc31a1* gene gradually increases from postnatal day 10 up to day 18 and its highest expression occurs between postnatal days 18 and 22, when the primary spermatocytes are the most abundant cells in the seminiferous tubules. In the testes of 30-day-old males, a significant reduction in *Slc31a1* gene expression was detected, indicating that it is lower when postmeiotic cells constitute a significant proportion of germ cells. This indicates that CTR1 supplies premeiotic and early meiotic germinal cells with copper necessary for maintenance of the meiosis process [65]. To examine the functional significance of CTR1 for the process of spermatogenesis, mice with conditional knock-out of the *Slc31a1* gene in the germinal cells (CTR1^ΔGC^ mice) were generated [64]. In CTR1^ΔGC^ males, a marked reduction in the size of the testes was observed in the postnatal days 28 and 41. Histological evaluation of testes in CTR1^ΔGC^ mice revealed that in the seminiferous tubules of 28-day-old males the number of primary pachytene spermatocytes was strongly reduced. The presence of germ cells with condensed nuclei in the testes of CTR1^ΔGC^ mice strongly suggested that primary spermatocytes died through apoptosis [64]. In the seminiferous tubules of the 41-day-old CTR1^ΔGC^ males, the majority of germ cells died, while spermatogonia and Sertoli cells were still present. All these data indicate that CTR1 expression in primary spermatocytes is critical for the process of spermatogenesis because it delivers large amounts of copper necessary for the correct course of meiotic division. Our results confirmed these observations and showed that during spermatogenesis progression in mouse gonads, copper concentration increases continuously, reaching the highest value at postnatal day 20, when meiotic germ cells are most prominent [65].

It is noteworthy that the *Ctr1* gene is conservative in eukaryotes. Both yeast (*Schizosaccharomyces pombe*, *S. pombe*) and insect (*Drosophila melanogaster*) models highlight the importance of the Ctr protein in the import of copper to germinal cells during the process of meiosis. In yeast, the four proteins Ctr4, Ctr5, Ctr6, and Mfc1 have been identified and characterized as copper transporters operating in meiotic cells. Among them, Ctr4, Ctr5, and Ctr6 are members of the CTR family of membrane copper transporters [66,67,68]. It has been shown that in *S. pombe* during meiosis the copper transporters Ctr4 and Ctr5 co-localize at the plasma membrane of zygotic cells shortly after the induction of meiosis under low copper conditions and play a critical role in the acquisition of this microelement from the environment [66,68]. In turn, Ctr6 localizes to vacuolar membranes in early meiosis and then undergoes redistribution in a time-dependent manner to reach forespore membranes where it persists until sporulation [66,68]. Ctr6 serves to mobilize intravacuolar stores of copper and participates in the delivery of copper to Sod1, a cytosolic Cu-dependent antioxidant enzyme in yeast [66,68]. In *S. pombe* the Mfc1 protein, a yeast homolog of mammalian CTR1, is necessary for the progression of meiotic cells through the first prophase. Importantly, functional similarities between yeast Mfc1 and mammalian CTR1 proteins have been demonstrated [69]. Mfc1 is produced throughout the meiotic division and spore maturation process. Mfc1 localizes in the surrounding of the forespore membrane and its presence is required for copper accumulation into forespores and production of fully active copper amine oxidases [66,69]. Cells with a deficiency of Mca1 protein, an activator of *Mfc1* gene transcription, are arrested at the first metaphase under Cu-deficient conditions [70]. Thus, it seems that a minimal copper level must be maintained to assure meiotic cell division in yeast. It is tempting to propose that a similar regulatory mechanism might act in mammalian meiotic cells.

In *Drosophila melanogaster* three *Ctr1-like* genes are expressed: *Ctr1A*, *Ctr1B*, and *Ctr1C*. The *Ctr1A* gene is constitutively and ubiquitously expressed. Its loss results in developmental arrest and in a general failure of Cu-dependent processes (cuproenzyme activity, neuropeptide maturation, heart function). The *Ctr1B* gene is responsible for copper uptake from the intestine. The *Ctr1C* gene has been proved to be necessary for copper transport into male germ cells. Results of immunohistochemical analysis revealed that its expression is restricted to the male germline cells. In the male gonads of wandering third instar larvae, *Ctr1C* expression has been found in spermatocyte cysts, and the Ctr1 immunopositive signal gradually became stronger from early to late spermatocyte cysts [71]. Confocal analysis showed that the Ctr1C protein localizes to the plasma membranes of late spermatocyte cysts but is absent from early cysts, and this indicates that copper is delivered to the male meiotic germ cells. In the gonads of mature *D. melanogaster* males, Ct1rC protein is localized in the mature spermatozoa. Interestingly, loss of function of the *Ctr1C* gene in *D. melanogaster* male mutants (*Ctr1C^6D^*) did not lead to infertility. However, double male mutants lacking functional Ctr1B and Ctr1C proteins, in which the intraorganismal copper level is strongly reduced due to the impairment of intestinal absorption of the metal (caused by a mutation in the *Ctr1B* gene) are sterile [71]. All these data attest to the requirement of CTR/Ctr proteins for copper transport to the male germ cells during spermatogenesis and show that this process is very conservative between yeast, insects, and mammals and that copper delivery to the developing male germ cells is critical for the finishing meiosis.

In the mouse testis, apart from germ cells, expression of the *Slc31a1* gene has also been reported in somatic cells such as Sertoli cells [19,64,65]. In the seminiferous tubules, Sertoli cells support, nourish, and protect spermatogenic cells using various signal pathways. In addition, tight junctions between adjacent Sertoli cells form a blood–testis barrier (BTB), building an environment optimal for the development of germ cells. The BTB regulates the free transport of metabolites, ions or harmful substances from entering the lumen of the seminiferous tubules and reaching the meiotic germ cells [63,64]. Immunohistochemical analysis has shown that CTR1 protein is localized along both the basal and adluminal compartments of the Sertoli cells [64,65]. To examine whether the loss of the *Slc31a1* gene expression in Sertoli cells affects spermatogenesis, mice with specific disruption of this gene in these cells (CTR1^ΔSC^ males) were generated. Interestingly, in CTR1^ΔSC^ males copper concentration in the testes was reduced by about 40% [64]. This strongly suggests that copper can be delivered to the seminiferous tubules via the Sertoli cells equipped with basally localized CTR1. Copper deficiency in the gonads of CTR1^ΔSC^ males also leads to decreased activity of cytochrome c oxidase by up to 60% compared to that in the testes of wild-type (WT) males [64]. Despite the reduction in copper concentration in the gonads of CTR1^ΔSC^ males, these animals were fertile. Moreover, when they were crossed with WT females to assess their fertility, no changes in the length of conception and the number of pups in the litter were observed [64]. In our studies, we also observed the CTR1 immunopositive signal in the lateral membrane of Sertoli cells, localized close to the lumen of seminiferous tubuli [65]. We hypothesized that such localization of CTR1 protein in the Sertoli cells allows reabsorption of Cu^+^ ions removed from the germinal cells by ATP7A and ATP7B copper transporters. The expression and function of the CTR1 protein in male mouse gonads is summarized in Table 1.

Little is known about the role of the CTR2 protein, a low-affinity cellular Cu^+^ ion importer involved in the maintenance of copper homeostasis in the gonads. To check whether this protein is also involved in copper import into primary spermatocytes, we analyzed the testicular temporal expression profile of the *Slc31a2* gene [65]. Results of the qRT-PCR analysis showed the lowest expression of this gene in the testes of 5- to 22-day-old males, but in 30-day-old and older animals, a significant increase in the *Slc31a2* gene expression was observed. Our findings indicate that this gene is predominantly expressed when postmeiotic cells are abundant in the testes of mice [65]. The role of the CTR2 protein during spermatogenesis needs future investigation.

## 6. Complex Regulation of the *Sod1* Gene Encoding Cu,Zn-Superoxide Dismutase (SOD1) in Male Gonads

For many years it has been believed that among various mammalian cell types, male germ cells are particularly vulnerable to excess copper [72,73]. Spermatogenesis is a process that consumes large amounts of oxygen. Testes are very susceptible to the reactive oxygen species due to the high content of unsaturated fatty acids in the membranes of testicular cells [74]. The ability of copper to react with partially reduced forms of O_2_ such as the superoxide anion (O_2_^−^) and hydrogen peroxide (H_2_O_2_) may lead to the formation of the hydroxyl radical (OH), a highly destructive oxidant [74,75]. The first enzyme involved in the protection against oxidative damage is SOD1, which catalyzes the conversion of O_2_^−^ to H_2_O_2_ and O_2_ dismutation. Then, H_2_O_2_ is metabolized by catalase (CAT) or, to a greater extent, by glutathione peroxidase (GPX) [74,76]. In mammalian somatic cells, SOD1 protein is responsible for approximately 90% of total SOD (superoxide dismutase) activity [75]. In the male gonads, the *Sod1* gene exhibits a specific and unusual expression pattern. In mouse testes, three alternative transcripts, (0.73, 0.80, and 0.93 kb), of the *Sod1* gene have been found [77]. In somatic cells of the gonad, the 0.73 kb *Sod1* (somatic, *sSod1*) transcript is synthesized whereas the 0.80 and 0.93 kb *Sod1* (testicular, *tSod1*) transcripts are specific only for the germ cells [77]. The *sSod1* and *tSod1* transcripts differ in 5′UTR (untranslated region on the 5’) structure [77]. The *tSod1* transcripts contain an additional 114 nucleotides in the 5′UTR as compared with the *sSod1* transcript [77].

In contrast to *sSod1*, *tSod1* is translationally regulated during spermatogenesis. In testes, SOD1 translation is repressed in meiotic cells and SOD1 protein was found only in spermatogonia [73]. It has been proposed by Gu and Hecht, 1996, that translation of 0.93 kb testicular *Sod1* transcript in the male gonads can be blocked by the 65 kDa protein, SOD-RBP (Cu,Zn-superoxide dismutase RNA-binding protein), which can be reversibly bound to the 5′UTR of the *tSod1* mRNAs [78]. The authors suggest that SOD-RBP recognizes a secondary structure formed by 114 additional nucleotides within the 5′UTR of *tSod1* [78]. However, expression and activity of SOD1 have been found in elongated spermatids and spermatozoa in humans, mice, and in other mammalian species [79,80,81,82,83,84]. Thus even if the expression of both 0.93 kb and, to a lesser extent, 0.80 kb *Sod1* mRNAs in round spermatids was detected [77,78], they are translationally repressed and stored until the elongated spermatid stage. For normal activity, SOD1 needs to undergo maturation from apo-protein and monomer form to a functional dimeric holo-enzyme. During this process, apo-SOD1 requires zinc and copper and the formation of an intramolecular disulfide bond between two cysteines to form a homodimer [28,85]. SOD1 maturation is facilitated by CCS, which catalyzes the acquisition of Cu ions and the formation of a disulfide bond [27,28]. Thus, CCS plays a crucial role not only in SOD1 metalation but also in SOD1 activation.

In the absence of metal cofactors and upon reduction of the disulfide bonds, the SOD1 dimer is destabilized and exists as an inactive monomer. Only when both metal ions are present and a disulfide bond is formed, the active, dimeric form of SOD1 is stabilized [27,28,85]. Analysis of the expression of the *Ccs* gene in the testes of young (immature) and adult (mature) mice revealed that its mRNA abundance in the testes of young males is relatively low in comparison to mature animals [65]. This can be explained by the fact that in the testes of young animals, *Ccs* is expressed mainly in somatic cells, in which the translation of *sSod1* takes place. Increased *Ccs* expression in the testes of adult males indicates that both *Sod1* transcripts (*sSod1* and *tSod1* in germ cells) are translated to support an efficient metalation of both apo-forms of SOD1 [65].

## 7. Role of ATP7A, Copper Transporting ATPase in the Protection of Premeiotic and Meiotic Germ Cells from Copper Toxicity

An important question arises as to the mechanism of preventing germinal cells (and in general the gonads) from the toxic effects of copper excess. A possible explanation has been provided by the analysis of the expression of Cu-transporting ATPases: ATP7A and ATP7B proteins in the mouse testes [65]. Cu-transporting ATPases are the only proteins that control cellular copper efflux in mammals. The expression levels of ATP7A and ATP7B are tissue- and organ-dependent and in some organs developmentally regulated [3,10,11,33,34]. In seminiferous tubules, ATP7A is expressed in the spermatocytes [65]. Quantitative analysis of the expression of the *Atp7A* gene and ATP7A protein in mouse testes showed that the highest expression level of both gene and protein occurs in males between days 18 and 22 postpartum, when primary spermatocytes are the most prominent germ cells [65,86]. In the testes of adult mice, positive staining was observed in leptotene and pachytene spermatocytes but not in earlier or later stages of germ cells. Interestingly, the ATP7A protein has not been detected in the round and elongated spermatids [65]. All these data strongly suggest that the function of ATP7A is associated with the efflux of excess copper from male germ cells at the first prophase (mainly pachytene spermatocytes) to protect them against free radicals potentially generated by Cu ions. Indeed, increased copper concentration is toxic for germ cells because it contributes to oxidative damage to DNA including DNA oxidation, single and double-strand breaks, crosslinks and adducts, point mutations, and chromosome instability [87]. However, it is evident that copper cannot be excessively exported from germ cells because its appropriate level is required for meiotic division [66,68]. Immunohistochemical studies have revealed that expression of ATP7A protein within seminiferous tubuli is not restricted to germ cells, but is also present in somatic Sertoli cells [65]. In the Sertoli cells, the ATP7A protein is localized close to the basolateral part of the seminiferous tubuli, indicating that this protein is involved in copper efflux possibly contributing to the protection of these cells from copper overload. Previous findings have also shown that in polarized cells, increased intracellular copper levels trigger relocalization of ATP7A to the basolateral cell membrane [3]. Localization of ATP7A in the Sertoli cell can suggest that in the testes, this protein participates in the regulation of copper concentration not only in germ cells but also in seminiferous tubules. As demonstrated in mice with mutations in the *Atp7a* gene, copper concentration in the testes is higher than in the wild genotype (WT) controls, and this finding supports the concept of ATP7A as a protein playing a role in the maintenance of copper homeostasis in the testes [48]. In general, our knowledge regarding ATP7A function originates mostly from studies on mouse models with different mutations within the X-linked *Atp7a* gene, called *mottled* mutants [12,88,89,90,91]. To examine the role of ATP7A protein in the process of spermatogenesis, we used mice with the *mosaic* mutation (*Atp7a^mo-ms^*) belonging to the group of mottled mouse models, recognized as animal models of Menkes disease [34,92]. In mosaic mice, the missense mutation c.2933G to C, in the *Atp7a* gene results in amino acid substitution Arg978Pro in the ATP7A protein [34]. Mosaic mutant males exhibit many clinical features characteristic for defective copper metabolism, including defects in pigmentation and hair structure, decrease in body weight, poor viability, and progressive paresis of the hind limbs. Similarly to other mottled mutants, mosaic mice exhibit disturbances in copper transport and absorption. In mutant males, copper is accumulated in the small intestine and kidneys, but the brain, liver, and heart have been found to be Cu-deficient [92,93,94]. Mutant males die at about days 16 postpartum, but if treated with subcutaneous injection of cupric chloride (CuCl_2_) solution, they survive longer and some of them achieve remarkable longevity [12,48]. Lack of activity of the ATP7A protein in mosaic mutants leads to the accumulation of copper in the testes, especially in mutant males treated with CuCl_2_ [48]. Copper accumulation in the gonads of the mutants results in increased apoptosis of the germ cells in both young and adult males [48,95]. In the testes of the adult mosaic mutants, apoptotic cells have frequently been identified among spermatocytes; for comparison, in mature testes of WT mice, sporadic apoptotic cell death is observed and apoptosis is largely restricted to spermatogonia [48,96]. This observation supports our findings showing that ATP7A activity in primary spermatocytes is of key importance for germ cells at the stage of the first prophase [65]. Histological analysis of the gonads from adult mutants has revealed gonad injury manifested as a presence of atrophic and vacuolated and in some males sclerotic seminiferous tubuli. Despite these abnormalities, spermatogenesis continues and mutant males produce spermatozoa [48,95]. Nevertheless, analysis of gamete quality has revealed a statistically significant decrease in the percentage of motile sperm, live spermatozoa, normal morphology, and spermatozoa tail membrane integrity in comparison with wild-type genotype control [48]. All the results confirm that ATP7A protein plays an important role in the process of spermatogenesis and lack of its activity leads to gonad injury and poor quality of gamete production. The expression and function of ATP7A protein in male mouse gonads is summarized in Table 1.

## 8. Role of the ATP7B Copper-Transporting ATPase in the Regulation of Copper Concentration in Postmeiotic Germ Cells

In the testes, in contrast to ATP7A, which is expressed in premeiotic and meiotic cells, ATP7B protein is located only in the postmeiotic germ cells—elongated spermatids. Moreover, ATP7B protein has been observed in elongated spermatids bound to Sertoli cells [65]. We postulate that in the elongated spermatids, ATP7B-bound copper in the Golgi apparatus can be transported with vesicles to the cytoplasm. This has been described for hepatocytes, in which increased copper concentration induces ATP7B trafficking from the TGN to a subset of lysosomes, where ATP7B imports copper into the lysosomal lumen and where the metal can be transiently stored [39]. It is possible that in elongated spermatids, the Cu-ATP7B complex is also transported to the lysosomal lumen and then removed from the cells with the residual cytoplasm, and can be engulfed by Sertoli cells [65]. In Sertoli cells, copper ions trapped in lysosomes might be taken up by the CTR2 transporter. Results of a quantitative analysis of the expression of the *Slc31a2* and *Atp7b* genes have revealed that its expression significantly increases in the testes of 30-day-old male mice in which the process of spermiogenesis has started [65].

In the testes, ATP7B protein has also been found outside of seminiferous tubules in the myoid cells surrounding seminiferous tubuli, where the presence of CP has also been localized [65]. We postulate that in the myoid cells, ATP7B plays a biosynthetic role and participates in the binding of Cu^+^ ions to the apo-CP. In mammalian cells, CP is produced in two forms resulting from alternative splicing of exons 19 and 20 of the *Cp* gene [97]. The shorter form of CP, called a secretory CP, is the main protein transporting copper all over the organism, and is mainly produced by hepatocytes and secreted to the blood [97,98]. In the longer form of CP, the last five amino acids are replaced by 30 alternative residues, leading to the addition of the GPI (Glycosylphosphatidylinositol) anchor [97]. This GPI-CP form is located in the plasma membrane and plays the role of ferroxidase, which catalyzes the oxidation of Fe^++^ to Fe^+++^ and acts in conjunction with the only known mammalian iron exporter (ferroportin (FPN)) to mediate iron release from the cells. CP has been shown to co-localize with FPN within the cell membrane, forming a complex that allows Fe ions to be exported to the extracellular environment and to be loaded on transferrin, which binds only Fe^+++^ ions [99,100].

In the testes, expression of GPI-CP has previously been found in the Sertoli cells and it has been suggested that GPI-CP is related to transepithelial iron transport [101]. This suggestion is confirmed by a recent study showing that in the Sertoli cells, ferroportin is located in the basal membrane and it can participate in iron export from the seminiferous tubules [102]. In seminiferous tubules, both iron and copper should be kept at a low, physiologically constant level to protect the developing germ cells from copper/iron-induced oxidative damage. In conclusion, we suggest that the function of ATP7B in myoid cells might be associated with CP function and iron transport.

Copper transport in the male gonads and localization of the Cu-transporter in the seminiferous tubule are summarized in Table 1 and Figure 2.

## 9. Summary

Both copper deficiency and overload in the gonads are important conditions, which can negatively influence spermatozoa quality and reproductive function in humans and many other mammalian species [47,48,49,50,53,54,64]. Results of experiments conducted on model organisms such as yeast, insects, and mice have revealed that during the process of gamete production an appropriate copper level is necessary for meiosis progression and subsequent cell reorganization to form mature spermatozoa. However, the increased copper level is toxic to germ cells. Therefore, organisms have developed tightly regulated molecular mechanisms responsible for adequate copper supply to the gonads and its transport to Cu-dependent enzymes, but also for the protection of testes from the toxic effects of copper overload.

## Figures and Tables

**Table 1:** The functions of copper-dependent proteins in the particular germ and somatic cells in the male mouse gonad.

	Cell Type	Germ Cells			Somatic Cells	
Protein		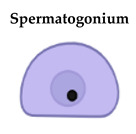	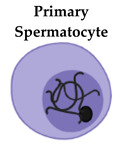	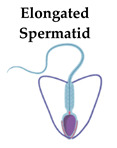	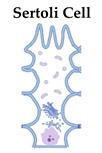	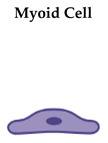
**CTR1**		Cu transport to the cell	Cu transport to the cell	Not found	Cu transport to the cell	Cu transport to the cell?
**ATP7A**		Not found	Cellular Cu efflux and protection of cells from copper overload	Not found	Cellular Cu efflux and protection of cells from copper overload	Not found
**ATP7B**		Not found	Not found	Cellular Cu efflux and protection of cells from copper overload	CP metalation	CP metalation
**SOD1**		Antioxidant protection	Not found	Antioxidant protection	Antioxidant protection	Not found
**CP**		Not found	Not found	Not found	Oxidation of Fe^++^ ions? Cu^+^ ions sequestering?	Oxidation of Fe^++^ ions
